# Ag85A DNA Vaccine Delivery by Nanoparticles: Influence of the Formulation Characteristics on Immune Responses

**DOI:** 10.3390/vaccines4030032

**Published:** 2016-09-12

**Authors:** Johanna Poecheim, Christophe Barnier-Quer, Nicolas Collin, Gerrit Borchard

**Affiliations:** 1 School of Pharmaceutical Sciences, University of Geneva-University of Lausanne, Rue Michel Servet 1, 1211 Geneva, Switzerland; johanna_poecheim@hotmail.com; 2 Vaccine Formulation Laboratory, Department of Biochemistry, University of Lausanne, Chemin des Boveresses 155, 1066 Epalinges, Switzerland; christophe.barnier-quer@unil.ch (C.B.-Q.); nicolas.collin@unil.ch (N.C.)

**Keywords:** DNA vaccine, nanoparticles, *N*-trimethyl chitosan, adjuvants, cell-mediated immunity, tuberculosis

## Abstract

The influence of DNA vaccine formulations on immune responses in combination with adjuvants was investigated with the aim to increase cell-mediated immunity against plasmid DNA (pDNA) encoding *Mycobacterium tuberculosis* antigen 85A. Different ratios of pDNA with cationic trimethyl chitosan (TMC) nanoparticles were characterized for their morphology and physicochemical characteristics (size, zeta potential, loading efficiency and pDNA release profile) applied in vitro for cellular uptake studies and in vivo, to determine the dose-dependent effects of pDNA on immune responses. A selected pDNA/TMC nanoparticle formulation was optimized by the incorporation of muramyl dipeptide (MDP) as an immunostimulatory agent. Cellular uptake investigations in vitro showed saturation to a maximum level upon the increase in the pDNA/TMC nanoparticle ratio, correlating with increasing Th1-related antibody responses up to a definite pDNA dose applied. Moreover, TMC nanoparticles induced clear polarization towards a Th1 response, indicated by IgG2c/IgG1 ratios above unity and enhanced numbers of antigen-specific IFN-γ producing T-cells in the spleen. Remarkably, the incorporation of MDP in TMC nanoparticles provoked a significant additional increase in T-cell-mediated responses induced by pDNA. In conclusion, pDNA-loaded TMC nanoparticles are capable of provoking strong Th1-type cellular and humoral immune responses, with the potential to be further optimized by the incorporation of MDP.

## 1. Introduction

Activation of potent Th1 cellular immune responses has been shown to play a role against *Mycobacterium tuberculosis* (*Mtb*) infection and reactivation. It is contemplated as a promising strategy for developing new effective vaccines against tuberculosis [1,2,3]. In order to achieve this, DNA vaccines offer an interesting approach. In situ expression of antigenic proteins encoded by plasmid DNA (pDNA) induce specific immune responses against the pathogen, without the risk of reversion into virulence associated with live-attenuated vaccines [4]. Moreover, DNA vaccines have the potential to induce both humoral and cellular immune responses, including cytotoxic T-cell responses, which is crucial to prevent the reactivation of latent tuberculosis infection and to mediate the elimination of intracellular *Mtb* [5].

Ag85A is among the major *Mtb* secretory proteins, and immune responses against this antigen play a key role in tuberculosis infection [6]. Accordingly, DNA encoding Ag85A is currently contemplated as a promising DNA vaccine candidate against *Mtb* due to its ability to stimulate humoral and cell-mediated immune responses in mice, as well as protection in mice against *Mtb* challenge [7]. However, limited biodistribution and low cellular uptake present significant limitations to DNA vaccine potency [8]. Therefore, in order to develop better DNA vaccine candidates, current research focuses on the use of appropriate adjuvant vehicles and systems, such as delivery vectors. Furthermore, some immunostimulatory adjuvants show potential to enhance immune responses against the encoded antigen and to modulate the outcome towards a Th1 bias and CD4+ T-cells’ activation [9].

Nanoparticles composed of cationic polymers, such as trimethyl chitosan (TMC), a chitosan derivative characterized by its permanent positive charges, can be loaded with pDNA by electrostatic interactions [10,11]. TMC nanoparticles have previously been shown to promote enhanced cellular uptake of nucleic acids and model antigens, as well as to induce dendritic cell maturation, which make them a potential candidate as an adjuvant for DNA vaccines [12,13].

Immune responses can be modulated by the activation of pattern recognition receptors of the innate immune system, such as Toll-like receptors (TLR) and NOD-like receptors. pDNA naturally contains unmethylated CpG islets for TLR-9 targeting that activate proinflammatory cytokine release upon receptor binding. Muramyl dipeptide (MDP), a ligand of NOD-like receptor 2 (NOD2), has been shown to enhance Th1 responses against leishmaniasis, AIDS/HIV and malaria in different animal models [14,15,16]. Co-delivery of pDNA with MDP by TMC nanoparticle transport allows simultaneous targeting of immune cells in order to potentiate Th1 immune responses in a synergistic fashion [17,18].

The aim of the present work was to investigate the influence of formulating a DNA vaccine encoding Ag85A with TMC nanoparticles, on the immune responses generated in mice. pDNA was adsorbed at the outer surface of these cationic nanoparticles, and the impact on the cell-mediated immune activation of different pDNA/TMC nanoparticle ratios was investigated. Investigations of these formulations were initiated with physicochemical characterization to better understand the adjuvant mechanism of TMC nanoparticles towards pDNA delivery, followed by in vitro uptake studies, with the goal to explore the correlation of the results to the outcome of the immunization studies. Finally, we aimed to optimize one pDNA/TMC nanoparticle formulation by co-delivery of MDP to further enhance cell-mediated immune responses in mice.

## 2. Materials and Methods

### 2.1. Materials

*N*-trimethyl chitosan with a degree of quaternization of 20% was synthesized from chitosan (ChitoClear cg10; Primex, Siglufjordur, Iceland). The methodology for the synthesis was adapted from Heuking et al., employing a one-step reductive methylation with methyl iodide for 70 min at 60 °C, in the presence of sodium hydroxide [19]. Chondroitin sulfate and bovine serum albumin (BSA, endotoxin <0.1 ng/mg) were obtained from Sigma-Aldrich (Buchs, Switzerland); UltraPure DNase/RNase-free distilled water and OptiMEM reduced serum medium were purchased from Life Technologies (Zug, Switzerland); muramyl dipeptide (MDP, Invivogen, San Diego, CA, USA) and the LabelIT Cy5 Nucleic Acid Labeling Kit (Mirus Bio LLC, Madison, WI, USA) were obtained from Labforce (Muttenz, Switzerland); paraformaldehyde (Alfa Aesar, Ward Hill, Haverhill, MA, USA); Triton X-100, phalloidin conjugated to Alexa 488 (Lonza, Basel, Switzerland); 4′,6-diamidino-2-phenylindole (DAPI; AppliChem, Axon Lab AG, Baden, Switzerland) used for cell staining. pDNA encoding for the Ag85A antigenic epitope of *Mtb* (V1Jns.tPA-85A, 5853 bp) was a kind gift from Institut Pasteur (Brussels, Belgium) and was amplified using the Endofree Qiagen plasmid Giga kit (Hombrechtikon, Switzerland). A549 cells (American Type Culture Collection, Rockville, MD, USA), a cell line derived from human carcinoma, were cultured in Ham’s F12 medium with 10% FCS, both from PAN Biotech GmbH (Aidenbach, Germany), and 1% penicillin/streptomycin (PS; Life Technologies, Zug, Switzerland). Mouse spleen cells were cultured in DMEM (Gibco), containing 10% fetal calf serum (FCS, Gibco), 1% PS, all obtained from Life Technologies, and 0.01% β-Mercaptoethanol and 2 mM glutamine, both from Sigma-Aldrich. The following reagent was obtained through BEI Resources, NIAID, NIH: Ag85A Recombinant Protein Reference Standard, NR-14871. 

### 2.2. Nanoparticle Preparation

Nanoparticles were formed according to the procedure described by Hansson et al. with some modifications [20]. Briefly, TMC and chondroitin sulfate were dissolved separately in water with 5 mg/mL TMC and 1 mg/mL chondroitin sulfate, and equal volumes of the two polymer solutions were mixed. The intermolecular linkages created between the positively-charged amino groups of TMC and the negatively-charged sulfate and carboxylate groups of chondroitin sulfate are responsible for the success of the nanoparticle formation process. The resulting nanoparticle dispersion was diluted 1:10 in phosphate-buffered saline (PBS). According to Endosafe^®^ (Charles River, Charleston, SC, USA) Test Record, nanoparticle suspensions showed an endotoxin level of below 1 EU/mL. In the case of MDP-containing nanoparticle formulations in the in vivo immunogenicity studies, 10 µg of the dipeptide per injection volume (50 µL) were added to the TMC solution before particle formation. If not stated otherwise, pDNA/TMC nanocomplexes were prepared according to the following procedure: pDNA in PBS, at four different concentrations, was added to the diluted nanoparticle suspension (15 µg TMC nanoparticles per 50 µL) while shaking, at concentrations of 1 µg, 10 µg, 50 µg and 100 µg per 50 µL, yielding pDNA to TMC nanoparticle weight ratios of 0.07 (pDNA/TMC-NPs-0.07), 0.7 (pDNA/TMC-NPs-0.7), 3 (pDNA/TMC-NPs-3) and 7 (pDNA/TMC-NPs-7), respectively. The formed nanocomplexes with adsorbed pDNA were kept at room temperature for a maximum of 1 h prior to experimental use.

### 2.3. Size and Zeta Potential

The particle size distribution was determined by means of dynamic light scattering (DLS) using a Malvern ZetaSizer Nano ZS (Malvern Instruments, Worcestershire, UK). The zeta potential (ζ) of the particles was measured by electrophoresis and laser Doppler velocimetry using the same equipment. Before the measurement, samples were diluted at a ratio of 1:10 in 1 mM sodium chloride. 

### 2.4. Loading Efficiency

The loading capacity of pDNA at different concentrations with a fixed amount of TMC nanoparticles was quantified by the separation of the nanoparticles from unloaded pDNA using centrifugal ultrafiltration (Vivaspin, Sartorius, Göttingen, Germany). Samples were centrifuged through 300-kDa polyethersulfone (PES) membranes at 3220× *g* for 20 min, and non-adsorbed pDNA in the filtrate was determined by measuring the absorption at a wavelength of 260 nm with an ND-1000 NanoDrop spectrophotometer (Thermo Scientific, Illkirch-Graffenstaden, France). Loading efficiency was expressed as a percentage as the amount of adsorbed pDNA compared to the amount of pDNA initially used to form the nanocomplexes. 

### 2.5. pDNA Release Profile

Nanoparticles with a pDNA/TMC nanoparticle ratio of 0.7, representing 100% pDNA adsorbed at the surface, were either diluted 1:10 in PBS (pH 7.4) or in 0.1 M phosphate-buffered solution (pH 4.5) and stored in several aliquots at 37 °C at 80 rpm in a shaking incubator (GFL Gesellschaft für Labortechnik, Burgwedel, Germany). At different time points after 1 h, 2 h, 3 h, 6 h, 8 h, 12 h and 24 h, the dispersions were placed in Vivaspin 300-kDa PES membrane tubes and centrifuged for 20 min at 3220× *g*. Released pDNA was measured spectrophotometrically in the filtrate at 260 nm. The absorption values for the filtrate of plain nanoparticles were used as a blank, and centrifugation of pDNA alone served as a positive control for 100% release. pDNA release was calculated using Equation (1):

(1) $$\mathrm{pDNA}\;\mathrm{release}\;(\%)\;=\;\frac{\left({\mathrm{OD}}_{\mathrm{pDNA}-\mathrm{NPs}}\;-\;{\mathrm{OD}}_{\mathrm{blank}}\right)}{{\mathrm{OD}}_{\mathrm{pDNA}}}\;\times \;100$$

### 2.6. Nanoparticle Uptake In Vitro

Cellular uptake of pDNA, covalently labeled with Cy5 (pDNA-Cy5), was imaged with an automated fluorescence microscope (ImageXpress Micro XL, Molecular Devices, Sunnyvale, CA, USA). A549 cells (10^5^ cells/mL) were seeded in a 96-well clear-bottom black BD Falcon plate (Becton Dickinson, Allschwil, Switzerland) with 100 µL per well and cultured at 37 °C and 5% CO_2_ for 24 h. Culture medium was replaced by PBS containing either pDNA, pDNA applied with Lipofectamine2000 3:1 (*v*/*w*) or the pDNA/TMC nanoparticle formulations. Exclusively in this study, pDNA was applied in the same concentration in all wells, to allow the comparability of the uptake abilities between pDNA alone and the pDNA/TMC nanoparticle formulations of different ratios, meaning different pDNA densities on the nanoparticle surfaces. Therefore, 2 µg of pDNA-Cy5 in PBS were added to varying concentrations of TMC nanoparticles while shaking, yielding pDNA to TMC nanoparticle weight ratios of 0.07 (pDNA-Cy5/TMC-NPs-0.07), 0.7 (pDNA-Cy5/TMC-NPs-0.7), 3 (pDNA-Cy5/TMC-NPs-3) and 7 (pDNA-Cy5/TMC-NPs-7). All samples, studied in triplicate in two separate experiments, were incubated with cells for 2 h followed by extensive washing (5 times with PBS) to remove material that remained at the external side of the cell membranes. The cells were then stained according to Hansson et al. [20]. Acquisition setup and image analysis was performed with MetaXpress software. Nine positions in the center of each 96-well sample were collected, and the data were averaged across three biological replicates. Following imaging analysis with MetaXpress software, data were evaluated by multi-wavelength scoring to calculate the percentage of Cy5-positive cells for each site within a well using Equation (2):

(2) $$\mathrm{Cells}\;\mathrm{with}\text{ pDNA-Cy5 }\mathrm{uptake}\;(\%)\;=\;\frac{\mathrm{Number}\;\mathrm{of}\;\mathrm{Cy}5\;\mathrm{positive}\;\mathrm{cells}}{\mathrm{Number}\;\mathrm{of}\;\mathrm{nuclei}\;\mathrm{count}}\;\times \;100$$

### 2.7. Immunization of Mice with pDNA-Loaded Nanoparticles

All animal studies were approved by the cantonal veterinary authority of Geneva, Switzerland (Service de la consommation et des affaires vétérinaires; Authorization Number 2475). Female C57BL/6 mice (Harlan, Itingen, Switzerland), 8 weeks old at the beginning of the experiment, were vaccinated 3 times by intramuscular (i.m.) injection (50 µL) in the thigh muscle, on Days 0, 21 and 42. To study the influence of pDNA dose on the outcome of immune responses, mice (*n* = 5) received increasing concentrations of pDNA adsorbed to a constant concentration of nanoparticles to obtain pDNA/TMC nanoparticle formulations of the ratios 0.07, 0.7, 3 and 7. In a follow-up experiment, each group of mice (*n* = 8) received pDNA/TMC-NPs-0.7 or pDNA/TMC-NPs-0.7 with 10 µg MDP per injection as an additional adjuvant. In each experiment, groups of mice were also immunized with pDNA alone in equivalent concentrations as in the TMC nanoparticle formulations, and PBS, respectively, as controls. Blood samples were collected through terminal cardiac puncture one week after the last injection. Sera, isolated by centrifugation, were stored at −20 °C before analysis. After sacrificing the animals, spleens were collected and immediately prepared for the immunological assays, antibody ELISA and IFN-γ ELISPOT. 

### 2.8. Antibody ELISA

Maxisorp Nunc-immunoplates (Nunc, Roskilde, Denmark) were coated with 1 µg/mL rAg85A in PBS overnight at 4 °C. To reduce unspecific binding, wells were blocked with 1% BSA (*w*/*v*) in PBS for 2 h at room temperature (RT). After extensive washing with PBS, serial dilutions of serum ranging from 100–2.2 × 10^5^ were applied in duplicates. After incubation for 1 h at RT and extensive washing, Ag85A-specific antibodies were detected using goat anti-mouse total IgG, IgG1 or IgG2c conjugated to horseradish peroxidase (Southern Biotech, Birmingham, AL, USA) for 1 h at ambient temperature and by developing plates with TMB substrate (3,3′,5,5′-tetramethylbenzidine substrate; (Becton Dickinson, Basel, Switzerland) for 5 min in the dark. Reactions were stopped by adding an equal volume of 1 N sulfuric acid, and the optical density (OD) was measured at 450 nm using an iMARK microplate absorbance reader (Bio-Rad Laboratories, Hercules, CA, USA). IgG2c/IgG1 ratios above unity, calculated from serum antibody titers, were associated with Th1-related immune response profiles. 

### 2.9. IFN-γ ELISPOT

MultiScreen HTS-HA filter plates (Merck Millipore, Saint Quentin en Yvelines, France) were coated overnight at 4 °C with anti-mouse IFN-γ antibody (BD Pharmingen, Basel, Switzerland), carefully washed five times with PBS and blocked for 2 h at 37 °C with DMEM medium containing 10% FCS. Single cell suspensions were prepared by passing the freshly-isolated spleens through 100-µm cell strainers and by treating the cells with ammonium chloride 0.84% for 1.5 min to lyse red blood cells. Splenocytes were plated in duplicate at 10^6^ cells, 5 × 10^5^ cells and 2.5 × 10^5^ cells per well and stimulated with 5 µg/mL rAg85A, 5 µg/mL ConA or media for 72 h at 37 °C. The plates were then washed with 0.05% PBS-Tween20 and incubated for 2 h at RT with biotinylated anti-mouse IFN-γ antibody (BD Pharmingen) diluted 1:100 in 0.05% PBS-Tween20 1% BSA. After washing the plates, streptavidin-alkaline phosphatase (Roche, Basel, Switzerland) was added to each well at 1 U/mL and incubated for 2 h at RT, washed and the filters developed using bromo-chloro-indolyl-phosphate/nitro blue tetrazolium ready-to-use (Sigma-Aldrich, St. Louis, MO, USA) until spots appeared. The reaction was stopped by washing the plates with deionized water. Plates were dried in the dark for several days, and spots were counted on an automated ELISPOT reader (Bioreader 2000; BioSys GmbH, Karben, Germany). Results were expressed as the number of IFN-γ-secreting cells (spots forming cells; SFC) per million cells, taking the cell dilution factor into consideration. 

### 2.10. Statistical Methods

Antibody titers were logarithmically transformed before statistical analysis. All data were analyzed by a two-tailed Mann–Whitney test to demonstrate significant differences between the experimental groups. The statistical analysis was carried out using Prism (Version 6, GraphPad, San Diego, CA, USA), and *p*-values lower than 0.05 were considered to be significant. 

## 3. Results

### 3.1. Nanoparticle Characterization

Hydrodynamic diameters, PDI values and zeta potential were assessed for the pDNA/TMC nanoparticle formulations prepared in PBS as applied in our in vivo immunogenicity study and in vitro cellular-uptake experiments, respectively, to ensure the comparability of the results for both preparation methods. Data were consistent between the preparation with constant TMC nanoparticles and varying pDNA concentrations and the preparation with fixed pDNA concentration and variable amounts of TMC nanoparticles (Table 1). Other than the final amount of each component used in the formulation composition, no differences in the physicochemical characteristics of the final product of pDNA/TMC nanoparticle formulations were observed. Hence, this allowed us to draw correlations between in vitro uptake and in vivo immunogenicity studies. Particle size measurements in PBS of unloaded TMC nanoparticles revealed hydrodynamic diameters of 527 nm, a PDI of 0.3 and zeta potentials of 10 mV. 

Upon pDNA adsorption, nanoparticles with pDNA/TMC nanoparticle ratios of 0.07 represented a size increase to approximately 700 nm, with stable polydispersity index (PDI) values of 0.3. However, surface charge dropped to near neutral values, indicative of charge neutralization through the electrostatic interaction of pDNA with the cationic nanoparticles. The formulation of pDNA/TMC-NPs-0.7 led to size contraction (406–436 nm) and zeta potential values below zero, of around −24 mV. By having further increased pDNA concentrations, forming pDNA/TMC-NPs-3, particle diameters remained at 401–485 nm, whereas surface charges decreased again to approximately −30 mV. For nanoparticles with the highest pDNA/TMC nanoparticle ratio (ratio of seven), larger hydrodynamic diameters were observed (519–652 nm); however, the zeta potential remained in a range between −22 and −36 mV. One hundred percent adsorption of pDNA at nanoparticles’ surfaces was observed for pDNA/TMC-NPS-0.07 and pDNA/TMC-NPS-0.7; while increasing the pDNA/TMC-NPs ratio induced lower adsorption rates for pDNA/TMC-NPS-3 and pDNA/TMC-NPS-7. Only 31% and 15% pDNA, respectively, of the initially-added pDNA were adsorbed. By extrapolating the quantity of pDNA from these percentages, it can be assumed that maximally 15 µg of pDNA are strongly adsorbed on these nanoparticles. 

### 3.2. Kinetics of pDNA Release

**Figure 1:**
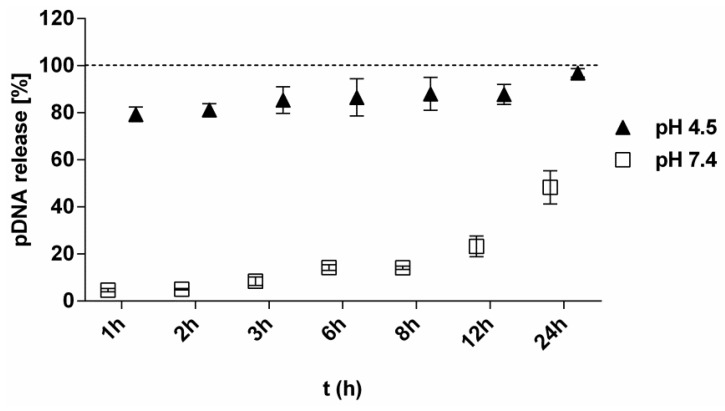
Release profiles of pDNA (10 µg) from pDNA/TMC-NPs-0.7 at pH 4.5 (▲) and pH 7.4 (□) at 37 °C for 24 h, calculated as a percent in relation to 100% pDNA in solution (dotted line). All measurement points are expressed as the means for a minimum of three measurements ± SEM.

In vitro release studies demonstrated highly pH-dependent release of pDNA from TMC nanoparticles. Significantly faster DNA release was observed at an acidic pH of 4.5 compared to physiological pH. The kinetics of pDNA release from TMC nanoparticles showed an initial burst release within 1 h at pH 4.5 (lysosomal pH) with 80% release, followed by 88% after 12 h and 100% release after 24 h (Figure 1). On the contrary, at physiological pH, pDNA/TMC nanoparticles remained stable for 8 h with only 14% pDNA release and dissociated slowly with only 50% pDNA being released after 24 h. 

### 3.3. Cellular Uptake

**Figure 2:**
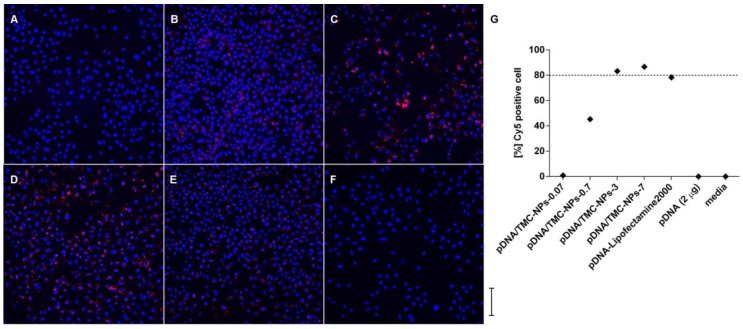
(**A**–**F**) Cellular uptake of Cy5-labeled pDNA (red) around nuclei (blue); (**G**) pDNA uptake was calculated for each well by calculating Cy5-positive cells (red) in relation to the number of nuclei (DAPI stained area; blue); representative wide field high content microscopy images of A549 cells, incubated for 2 h with formulations of pDNA-Cy5/TMC-NPs-0.07 (1% uptake; (A)); pDNA-Cy5/TMC-NPs-0.7 (45% uptake; (B)); pDNA-Cy5/TMC-NPs-3 (83% uptake; (C)); pDNA-Cy5/TMC-NPs-7 (87% uptake; (D)); pDNA-Cy5-Lipofectamine2000 (78% uptake; (E)); and pDNA-Cy5 alone (0% uptake; (F)). Images were taken at 20× magnification, and the scale bar represents 500 µm. All samples were studied in triplicate in two separate experiments.

The formulations for in vitro uptake were prepared with fixed pDNA-Cy5 quantities and decreasing concentrations of TMC nanoparticles, in order to obtain the same ratios of labeled pDNA in relation to TMC nanoparticles as in the in vivo experiments, rising from 0.07, 0.7, 3 to 7. This was thought necessary to allow the comparability and quantification of pDNA-Cy5 uptake for the different formulations applied. Cell viability for pDNA/TMC nanoparticle formulations using unlabeled pDNA was shown to be above 80% (Supplementary Material Figure S1). It was found that the level of uptake was the highest for pDNA-Cy5/TMC-NPs-3 and pDNA-Cy5/TMC-NPs-7, calculated from Figure 2A–F and visualized in Supplementary Material Figure S2. As shown in Figure 2G, 87% of Cy5-positive cells were detected after incubation with pDNA-Cy5/TMC-NPs-7 and 83% with pDNA-Cy5/TMC-NPs-3. Cellular uptake of pDNA-Cy5/TMC-NPs-0.7 was notably reduced to 45%, and for pDNA-Cy5/TMC-NPs-0.07 only 1% of Cy5-positive cells were found. No pDNA uptake was observed for pDNA-Cy5 in the absence of nanoparticle delivery. 

### 3.4. Immunogenicity of Different pDNA/TMC Nanoparticle Ratios

**Figure 3:**
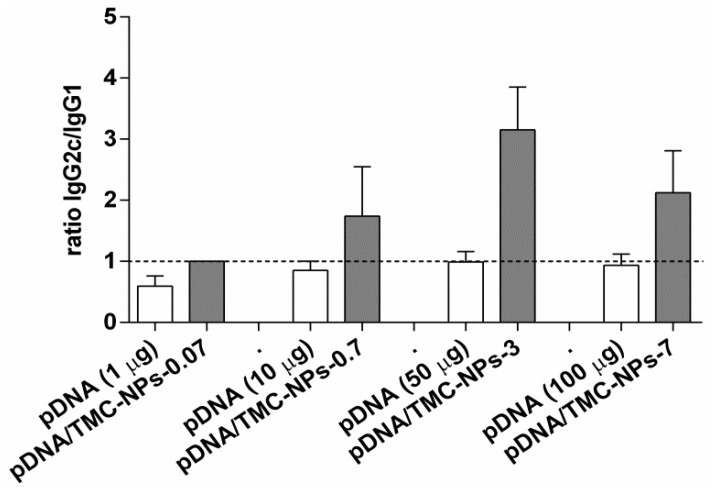
Influence of different pDNA doses on Th1 and Th2 polarization, either alone or adsorbed to TMC nanoparticles. Mice were immunized three times at three-week intervals, and sera were analyzed by ELISA at Week 7. Bars represent the mean (*n* = 5) ± SEM. The ratios of IgG2c and IgG1 antibodies specific to Ag85A were determined, and values higher than one (dotted line) characterize Th1-biased immune responses.

To study the influence of pDNA dose on the outcome of immune responses, mice were immunized with increasing concentrations of pDNA (1 µg, 10 µg, 50 µg and 100 µg) adsorbed to a constant concentration of nanoparticles to obtain pDNA/TMC nanoparticle formulations of the ratios of 0.07, 0.7, 3 and 7. High IgG2c titers are associated with Th1-mediated immune responses and IgG1 titers with Th2-type responses. As shown in Figure 3, the IgG2c/IgG1 ratios of the lowest dose of pDNA applied were below unity. Thus, the high levels of IgG1 induced by low-dose pDNA are in favor of the Th2 responses. These responses were altered by TMC nanoparticles (pDNA/TMC-NPs-0.07) to mixed Th1/Th2 responses, seen in the increase of IgG2c titers and the decrease of IgG1 titers (Supplementary Material Figure S3). With higher pDNA/TMC nanoparticle ratios (ratios of 0.7, 3 and 7), which implies higher pDNA content adsorbed to TMC nanoparticles, a clear shift to Th1-type immune responses could be induced. This increase of Th1-biased immune responses was observed with a maximum effect for pDNA/TMC-NPs-3. Interestingly and unanticipatedly, when having further increased pDNA concentrations and, hence, having further increased the ratio of pDNA to TMC nanoparticles to seven, the Th1-biased immune responses waned. 

### 3.5. Impact of MDP on the pDNA/TMC-NPs-0.7 Formulation

**Figure 4:**
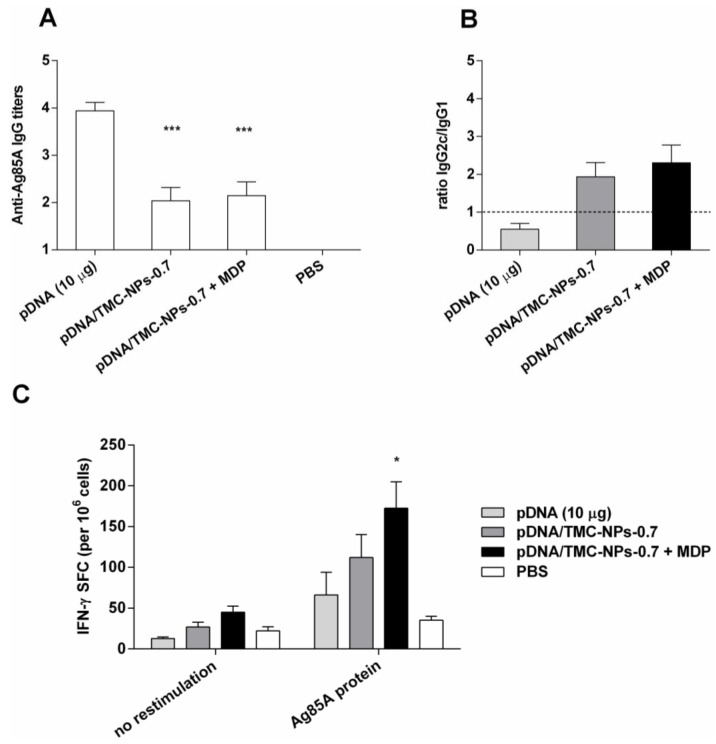
Immune responses in mice one week after the second booster injection (intramuscularly). (**A**–**C**) pDNA (10 µg) formulations ± MDP (10 µg) and/or TMC nanoparticles per dose. Bars represent the mean *n* = 8 ± SEM, * *p* < 0.05; *** *p* < 0.001 compared to pDNA alone. (A) Ag85A-specific serum IgG titers, analyzed by endpoint ELISA; (B) corresponding average Log IgG2c/ Log IgG1 ratios, indicative for the quality of the immune response; (C) IFN-γ secretion in mouse splenocytes analyzed by ELISPOT and data expressed as spot-forming cell (SFC) responses to media (no restimulation) and Ag85A protein.

The formulation with the lowest ratio (pDNA/TMC-NPs-0.7) capable of polarizing immune responses to a Th1 bias, as shown in Figure 3, was chosen to investigate the impact of MDP addition on enhancing cell-mediated immune responses. Ten micrograms of MDP per dose were applied (weight ratio to pDNA of 1:1 per formulation). This complies with previously published MDP dosages in adjuvant combination studies already tested in vivo [21,22]. pDNA was adsorbed onto TMC particles (pDNA/TMC-NPs-0.7) or applied with MDP containing TMC particles (pDNA/TMC-NPs-0.7 + MDP). An immunopotentiating effect in vivo was sought to be investigated, as we have reported in our laboratory a synergistic enhancement of proinflammatory cytokine release in vitro by co-delivery of pDNA with MDP [23]. As these in vitro experiments did not demonstrate any macrophage activation with MDP only [23], we excluded this group from the animal experiments. As shown in Figure 4A, significantly lower Ag85A-specific total IgG antibody production was detected following vaccination with both adjuvanted pDNA formulations (*p* < 0.001), pDNA/TMC-NPs-0.7 and pDNA/TMC-NPs-0.7 + MDP, when compared to vaccination with naked pDNA. Similar results were observed for the IgG1 isotype (Supplementary Material Figure S4). However, IgG2c isotype levels were significantly increased by pDNA delivered with TMC nanoparticles (*p* < 0.05) and were even more enhanced with MDP (*p* < 0.01), compared to the non-adjuvanted pDNA. To characterize the polarization of immune responses induced by the different vaccine formulations, ratios of IgG2c/IgG1 titers were calculated from ELISA analyses of mouse sera, as shown in Figure 4B. The major important difference between the effects of adjuvanted and non-adjuvanted pDNA appeared to be the type of immune response elicited. Mice vaccinated with naked pDNA developed marked Th2 immune responses with high IgG1 titers. IgG2c/IgG1 ratios were shifted to a value above unity for the pDNA/TMC nanoparticles, as well as for the pDNA/TMC-MDP nanoparticles, which is associated with a Th1-type immune response. 

Finally, the number of IFN-γ-producing spleen cells after restimulation with the antigen was determined by ELISPOT (Figure 4C) and confirmed the Th1 polarizing effect of the adjuvanted pDNA formulations. The numbers of IFN-γ-producing T-cells were shown to be enhanced by TMC nanoparticles. MDP in the pDNA/TMC nanoparticle formulation further increased the Th1 increasing effect of pDNA/TMC nanoparticles, resulting in significant higher cell-mediated responses compared to non-adjuvanted pDNA (*p* < 0.05). 

## 4. Discussion

The investigation of the physicochemical properties and in vitro characterization of nanoparticle-based DNA vaccines provide a potential context for the interpretation of the effects of formulation parameters on the type and extent of immune response elicited. In this study, the zeta potential of the particles with the lowest pDNA/TMC nanoparticle ratio was neutral, whereas all of the other pDNA/TMC nanoparticle formulations showed a negative zeta potential. Different doses of pDNA in the TMC nanoparticle formulation presumably lead to variable packing densities of pDNA at the surface of the nanoparticles. According to the loading efficiency studies, 15 µg of pDNA is the maximum amount strongly bound to the nanoparticles. pDNA/TMC-NPs-3 with 50 µg pDNA and pDNA/TMC-NPs-7 with 100 µg pDNA per injection dose contain excess amounts of pDNA that is either loosely bound (conceivably in pDNA/TMC-NPs-3) or remains as free unbound pDNA in suspension (pDNA/TMC-NPs-7). Surplus pDNA may be easily dissociated due to repulsion as a consequence of higher pDNA densities on the nanoparticle surfaces or may partly stay in suspension. The pDNA/TMC nanoparticle complexes were shown to be sensitive to pH and are particularly appealing for targeted intracellular release. While maintaining their electrostatically-conjugated structure in the physiological environment after injection, to efficiently transfer pDNA into the host cell, pDNA can be liberated from the nanoparticles inside lysosomes at pH 4–5 after endocytosis to allow entrance into the nucleus for gene transcription.

The TMC nanoparticle concentration varied in cellular uptake experiments and was the highest for pDNA/TMC-NPs-0.07. However, DNA uptake was shown to be the lowest for this formulation. pDNA/TMC-NPs-0.07 display a neutral surface charge and, consequently, seem to resist interaction with cells showing minimal internalization. It is well-known that cationic particles interact with negatively-charged cell membranes and are therefore very efficient in cell membrane penetration. Yet, we could show that despite their negative surface coating, pDNA/TMC nanoparticles of ratios 0.7, 3 and 7 were readily taken up by cells. Similar events of uptake of negatively-charged particles have also been reported by others [24,25]. Giljohann et al. quantified the cellular uptake of oligonucleotide loaded nanoparticles, having observed that higher densities of DNA on the surface lead to greater uptake, which is in accordance with our studies [26]. Their investigations revealed that the nanoparticles adsorbed serum proteins on the surface through electrostatic and hydrophobic interactions. The amount of proteins increased with the amount of oligonucleotides on the particle surface, and their uptake into cells might be due to the amount and nature of the proteins that are bound to the DNA strands. These investigations let us assume that the amount of pDNA present on the nanoparticle surface might influence the uptake of our pDNA/TMC nanoparticle formulations, rather than the quantity of nanoparticles. 

Looking closer at the serum IgG antibody subclasses, free pDNA in all concentrations induced high IgG1 antibody titers (Supplementary Material Figure S3). The non-adsorbed pDNA may provoke the induction of humoral immunity and suppress IgG2c formation associated with Th1 cell-mediated responses. The high IgG1 titers, as well as high total IgG levels detected in mice immunized with naked pDNA may be caused by the presence of DNA that remains at the outside of cells after i.m. injection. It has been suggested that the presence of extracellular host DNA, released from necrotic cells exposed to alum, acts as a damage-associated molecular pattern and is linked to alum adjuvant activity, inducing a Th2 bias of the subsequent adaptive response [27]. Extracellular DNA represents a condition not to be found in healthy subjects and, thus, may stimulate the immune system, offering a “danger signal”, and strongly boost the response in a similar way as proposed for alum adjuvant activity. TMC nanoparticles changed the impact of pDNA on serum IgG antibody responses towards Th1-related immunity. This might be partly caused by the nanoparticles themselves, as chitosan and trimethyl chitosan particles reportedly provoke dendritic cell maturation and proinflammatory cytokine production by phagocytosis-dependent mechanisms [28,29]. Furthermore, higher quantities of ingested pDNA due to TMC nanoparticle delivery may lead to stronger intracellular stimulation of endosomal TLR9 via unmethylated CpG sequences found in pDNA, as well as cytosolic TANK-binding kinase-1 activation by the double-stranded B-form of pDNA. Both incitements activate NF-κB to promote proinflammatory gene transcription and, thus, Th1 cell differentiation [30]. 

The adjuvant activity of MDP in combination with pDNA may be attributed to a cross-talk between NOD2 and the TLR-9 receptor pathways activating NF-κB in a synergistic fashion [17]. Several studies reported a synergistic outcome on inflammatory cytokine expression in different cells stimulated with NLR and TLR agonist in parallel, having observed synergy between TLR-2, TLR-4 and TLR-9 agonists and both NOD1 and NOD2 elicitors in macrophages and epithelial cells [31,32,33]. In terms of biological relevance, activation of both TLRs and NLRs might be important for the fine-tuning of the inflammatory response and was successfully applied in this study in co-delivery of those ligands with TMC nanoparticles. 

## 5. Conclusions

Our studies provided evidence that the combination of adjuvants to Ag85A-encoding pDNA in a single nanoparticle formulation can lead to an effective targeting of the cellular immune system and developed profound Th1 cell-mediated immune responses in mice. We believe that the information gained from the investigation of the physicochemical properties and in vitro characterization of nanoparticle-based pDNA formulations is valuable for the design of effective DNA vaccines. Cellular uptake investigations of pDNA in vitro showed saturation to a maximum level upon the increase in pDNA/TMC nanoparticle ratios, correlating with increasing Th1-related antibody responses up to a definite ratio. Variations in pDNA doses changed the outcome of immune responses and indicated that dose investigation is crucial for the desired outcome of Th1-biased immune responses. Hence, a potential context for the interpretation of the effects of formulation parameters on the type and extent of immune response elicited in vivo can be provided. An adjuvant effect of MDP co-delivered with pDNA/TMC nanoparticles was demonstrated in a further increase in Th1-associated antibody levels, as well as in the numbers of IFN-γ-producing T-cells. Such responses, which correlate with cell-mediated immune responses, are critical for the control of intracellular pathogens and may render pDNA/TMC nanoparticles a potential candidate for further investigations of protective efficacy against *Mtb* infections in a challenge model.

## Figures and Tables

**Table 1:** Particle size (Z-av), polydispersity index (PDI), zeta potential (ζ) and loading efficiency (LE) of trimethyl chitosan (TMC) nanoparticles (NPs) with or without pDNA (*n* = 3). pDNA/TMC-NPs were either prepared with constant TMC nanoparticles and varying pDNA concentrations or with fixed pDNA concentration and variable amounts of TMC nanoparticles.

Sample Name	Fixed TMC-NP Concentration			Fixed pDNA Concentration		
	Z-av (nm)	PDI	ζ (mV)	Z-av (nm)	PDI	ζ (mV)
TMC-NPs unloaded	527 ± 17	0.3	10 ± 1			
pDNA/TMC-NPs-0.07	684 ± 107	0.3	2 ± 0.4	712 ± 58	0.3	1 ± 1
pDNA/TMC-0.7	406 ± 3	0.3	−24 ± 2	436 ± 14	0.3	−23 ± 3
pDNA/TMC-3	485 ± 8	0.4	−28 ± 2	401 ± 20	0.3	−30 ± 3
pDNA/TMC-7	652 ± 26	0.4	−36 ± 1	519 ± 8	0.4	−22 ± 4

## Supplementary Materials

The following figures are available online at www.mdpi.com/2076-393X/4/3/32/s1. Figure S1: Cell viability of A549 cells; Figure S2: Cellular uptake of different pDNA-Cy5 formulations into A549 cells; Figure S3: Anti-Ag85A antibody responses (IgG, IgG1, IgG2c) in mice vaccinated with different pDNA doses, applied either alone or adsorbed to TMC nanoparticles; Figure S4: IgG1 and IgG2c isotypes from sera were determined by ELISA.

## References

[1] Hoft D.F., Kemp E.B., Marinaro M., Cruz O., Kiyono H., McGhee J.R., Belisle J.T., Milligan T.W., Miller J.P., Belshe R.B. (1999). A double-blind, placebo-controlled study of mycobacterium-specific human immune responses induced by intradermal bacille calmette-guérin vaccination. J. Lab. Clin. Med..

[2] Lindblad E.B., Elhay M.J., Silva R., Appelberg R., Andersen P. (1997). Adjuvant modulation of immune responses to tuberculosis subunit vaccines. Infect. Immun..

[3] Tameris M., Geldenhuys H., Luabeya A.K., Smit E., Hughes J.E., Vermaak S., Hanekom W.A., Hatherill M., Mahomed H., McShane H. (2014). The candidate TB vaccine, MVA85A, induces highly durable Th1 responses. PLoS ONE.

[4] Ingolotti M., Kawalekar O., Shedlock D.J., Muthumani K., Weiner D.B. (2010). DNA vaccines for targeting bacterial infections. Expert Rev. Vaccines.

[5] Kutzler M.A., Weiner D.B. (2008). DNA vaccines: Ready for prime time?. Nat. Rev. Genet..

[6] Yuk J.-M., Jo E.-K. (2014). Host immune responses to mycobacterial antigens and their implications for the development of a vaccine to control tuberculosis. Clin. Exp. Vaccine Res..

[7] Huygen K., Denis O., Montgomery D.L., Yawman A.M., Deck R.R., DeWitt C.M., Orme I.M., Baldwin S., D’Souza C., Drowart A. (1996). Immunogenicity and protective efficacy of a tuberculosis DNA vaccine. Nat. Med..

[8] Dupuis M., Denis-Mize K., Woo C., Goldbeck C., Selby M.J., Chen M., Otten G.R., Ulmer J.B., Donnelly J.J., Ott G. (2000). Distribution of DNA vaccines determines their immunogenicity after intramuscular injection in mice. J. Immunol..

[9] Kim J.J., Simbiri K.A., Sin J.I., Dang K., Oh J., Dentchev T., Lee D., Nottingham L.K., Chalian A.A., Mccallus D. (1999). Cytokine molecular adjuvants modulate immune responses induced by DNA vaccine constructs for HIV-1 and SIV. J. Interferon Cytokine Res..

[10] Amidi M., Romeijn S.G., Borchard G., Junginger H.E., Hennink W.E., Jiskoot W. (2006). Preparation and characterization of protein-loaded *N*-trimethyl chitosan nanoparticles as nasal delivery system. J. Control. Release.

[11] Thanou M.M., Verhoef J.C., Romeijn S.G., Nagelkerke J.F., Merkus F.W.H.M., Junginger H.E. (1999). Effects of *N*-trimethyl chitosan chloride, a novel absorption enhancer, on Caco-2 intestinal epithelia and the ciliary beat frequency of chicken embryo trachea. Int. J. Pharm..

[12] Slütter B., Plapied L., Fievez V., Alonso Sande M., des Rieux A., Schneider Y.-J., van Riet E., Jiskoot W., Préat V. (2009). Mechanistic study of the adjuvant effect of biodegradable nanoparticles in mucosal vaccination. J. Control. Release.

[13] Thanou M., Florea B., Geldof M., Junginger H., Borchard G. (2002). Quaternized chitosan oligomers as novel gene delivery vectors in epithelial cell lines. Biomaterials.

[14] Bomford R., Stapleton M., Winsor S., McKnight A., Andronova T. (1992). The control of the antibody isotype response to recombinant human immunodeficiency virus gp120 antigen by adjuvants. AIDS Res. Hum. Retrovir..

[15] Lemesre J.-L., Holzmuller P., Gonçalves R.B., Bourdoiseau G., Hugnet C., Cavaleyra M., Papierok G. (2007). Long-lasting protection against canine visceral leishmaniasis using the LiESAp-MDP vaccine in endemic areas of france: Double-blind randomised efficacy field trial. Vaccine.

[16] Pye D., Vandenberg K.L., Dyer S.L., Irving D.O., Goss N.H., Woodrow G.C., Saul A., Alving C.R., Richards R.L., Ballou W.R. (1997). Selection of an adjuvant for vaccination with the malaria antigen, MSA-2. Vaccine.

[17] Abbott D.W., Yang Y., Hutti J.E., Madhavarapu S., Kelliher M.A., Cantley L.C. (2007). Coordinated regulation of toll-like receptor and NOD2 signaling by K63-linked polyubiquitin chains. Mol. Cell. Biol..

[18] Higgins S.C., Mills K.H. (2010). TLR, NLR agonists, and other immune modulators as infectious disease vaccine adjuvants. Curr. Infect. Dis. Rep..

[19] Heuking S., Adam-Malpel S., Sublet E., Iannitelli A., Stefano A.d., Borchard G. (2009). Stimulation of human macrophages (Thp-1) using toll-like receptor-2 (TLR-2) agonist decorated nanocarriers. J. Drug Target..

[20] Hansson A., di Francesco T., Falson F., Rousselle P., Jordan O., Borchard G. (2012). Preparation and evaluation of nanoparticles for directed tissue engineering. Int. J. Pharm..

[21] Cheng C., Jain P., Bettahi I., Pal S., Tifrea D., Luis M. (2011). A TLR2 agonist is a more effective adjuvant for a chlamydia major outer membrane protein vaccine than ligands to other TLR and NOD receptors. Vaccine.

[22] Moschos S.A., Bramwell V.W., Somavarapu S., Alpar H.O. (2005). Comparative immunomodulatory properties of a chitosan-MDP adjuvant combination following intranasal or intramuscular immunisation. Vaccine.

[23] Poecheim J., Barnier Quer C., Heuking S., Brunner L., Collin N., Borchard G. (2015). Nanocarriers for DNA vaccines: Co-delivery of TLR-9 and NLR-2 leads to synergistic enhancement of proinflammatory cytokine release. Nanomaterials.

[24] Limbach L.K., Li Y., Grass R.N., Brunner T.J., Hintermann M.A., Muller M., Gunther D., Stark W.J. (2005). Oxide nanoparticle uptake in human lung fibroblasts: Effects of particle size, agglomeration, and diffusion at low concentrations. Environ. Sci. Technol..

[25] Patil S., Sandberg A., Heckert E., Self W., Seal S. (2007). Protein adsorption and cellular uptake of cerium oxide nanoparticles as a function of zeta potential. Biomaterials.

[26] Giljohann D.A., Seferos D.S., Patel P.C., Millstone J.E., Rosi N.L., Mirkin C.A. (2007). Oligonucleotide loading determines cellular uptake of DNA-modified gold nanoparticles. Nano Lett..

[27] Marichal T., Ohata K., Bedoret D., Mesnil C., Sabatel C., Kobiyama K., Lekeux P., Coban C., Akira S., Ishii K.J. (2011). DNA released from dying host cells mediates aluminum adjuvant activity. Nat. Med..

[28] Babensee J.E. (2008). Interaction of dendritic cells with biomaterials. Semin. Immunol..

[29] Bal S.M., Slütter B., van Riet E., Kruithof A.C., Ding Z., Kersten G.F.A., Jiskoot W., Bouwstra J.A. (2010). Efficient induction of immune responses through intradermal vaccination with *N*-trimethyl chitosan containing antigen formulations. J. Control. Release.

[30] Cui Z., Mumper R.J. (2003). Microparticles and nanoparticles as delivery systems for DNA vaccines. Crit. Rev. Ther. Drug Carr. Syst..

[31] Tada H., Aiba S., Shibata K.-I., Ohteki T., Takada H. (2005). Synergistic effect of Nod1 and Nod2 agonists with toll-like receptor agonists on human dendritic cells to generate interleukin-12 and T helper type 1 cells. Infect. Immun..

[32] Uehara A., Sugawara Y., Kurata S., Fujimoto Y., Fukase K., Kusumoto S., Satta Y., Sasano T., Sugawara S., Takada H. (2005). Chemically synthesized pathogen-associated molecular patterns increase the expression of peptidoglycan recognition proteins via toll-like receptors, Nod1 and Nod2 in human oral epithelial cells. Cell. Microbiol..

[33] Van Heel D.A., Ghosh S., Butler M., Hunt K., Foxwell B.M.J., Mengin-Lecreulx D., Playford R.J. (2005). Synergistic enhancement of toll-like receptor responses by nod1 activation. Eur. J. Immunol..

