# Supplementary material for: Ag85A DNA Vaccine Delivery by Nanoparticles: Influence of the Formulation Characteristics on Immune Responses
Source: Vaccines (Basel). 2016 Sep 12;4(3):32. doi: 10.3390/vaccines4030032 (PMC5041026; doi:10.3390/vaccines4030032)
Supplement: Supplementary file 1 [file vaccines-04-00032-s001.docx]

Supplementary Materials: Ag85A DNA Vaccine Delivery by Nanoparticles: Influence of the Formulation Characteristics on Immune Responses

Johanna Poecheim, Christophe Barnier-Quer, Nicolas Collin and Gerrit Borchard





**Figure S1.** Cell viability of A549 cells.


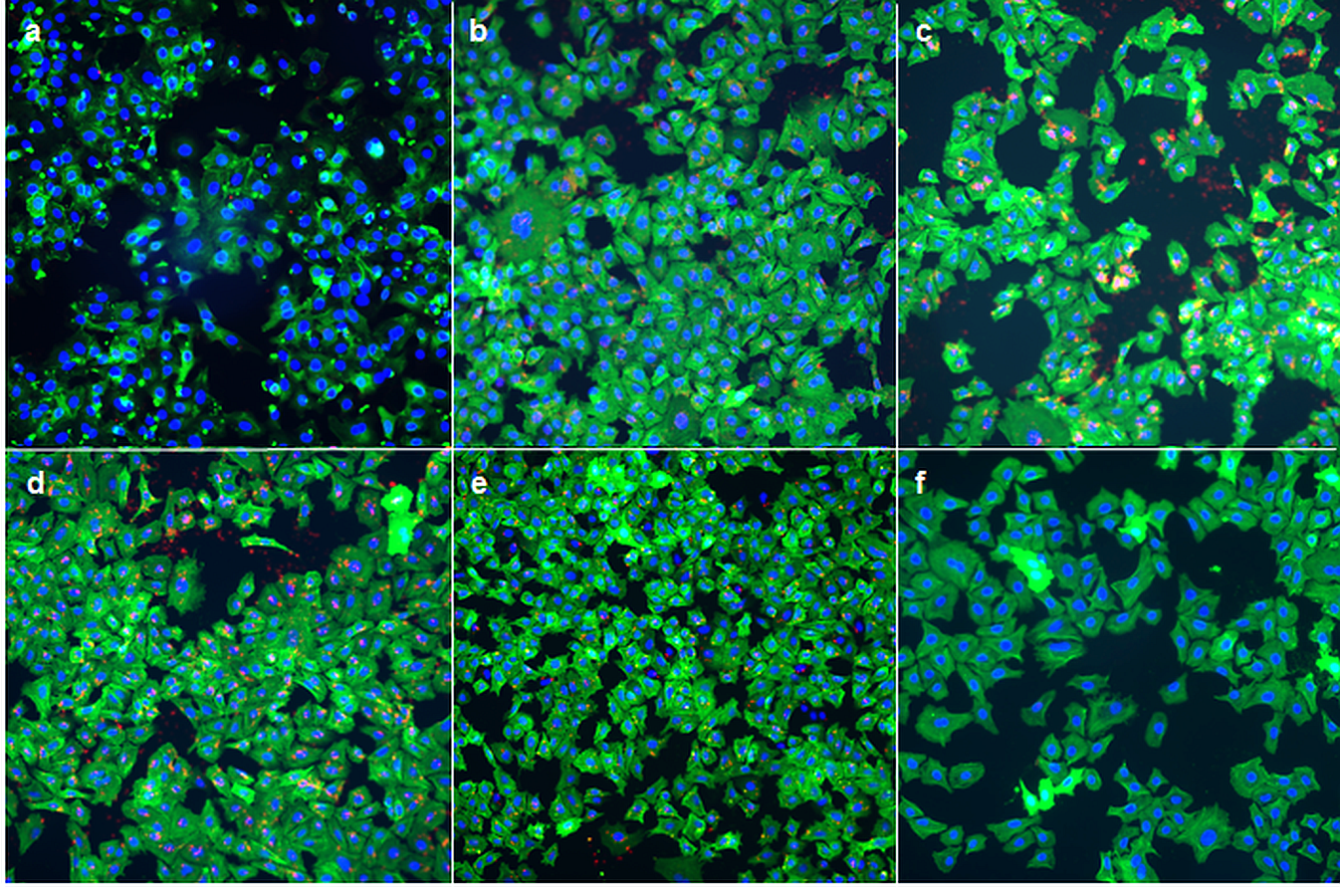


**Figure S2.** Cellular uptake of different pDNA-Cy5 formulations into A549 cells.





**Figure S3.** Anti-Ag85A antibody responses (IgG, IgG1, IgG2c) in mice vaccinated with different pDNA doses, applied either alone or adsorbed to TMC nanoparticles.





**Figure S4.** IgG1 and IgG2c isotypes from sera were determined by ELISA.
